# Supplementary material for: Impacts of environmental conditions, and allelic variation of cytosolic glutamine synthetase on maize hybrid kernel production
Source: Commun Biol. 2021 Sep 17;4:1095. doi: 10.1038/s42003-021-02598-w (PMC8448750; doi:10.1038/s42003-021-02598-w)
Supplement: Supplementary file 2 — Supplementary Information [file 42003_2021_2598_MOESM2_ESM.pdf]

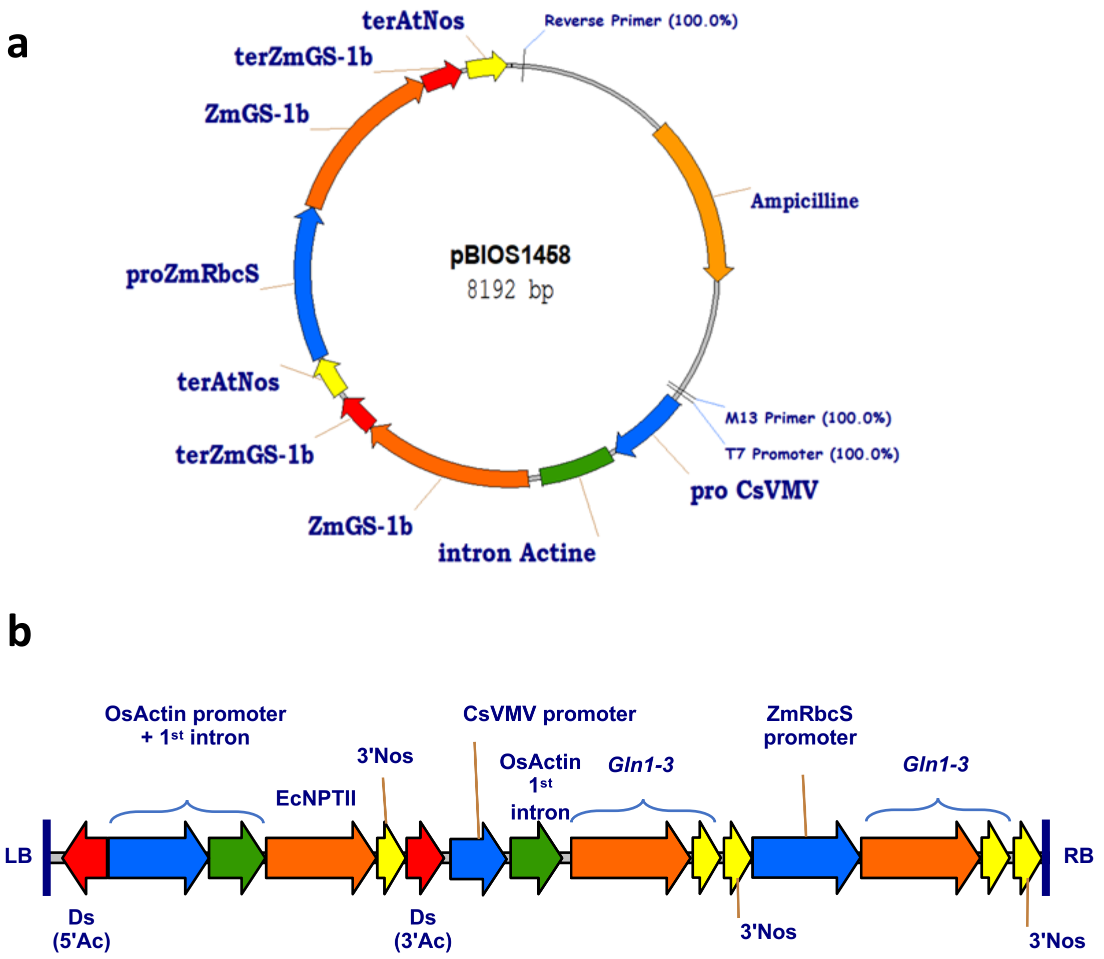

Supplementary Data Fig. 1: Map of the plasmids pBIOS1458 and pBIOS1459 containing the *Gln1-3* cDNA under the control of the cassava vein mosaic virus promoter (*CsVMV*) and the maize promoter (*RbcS)*. (**a**) Plasmid pBIOS 1458 containing the *Gln1-3* cDNA under the control of the cassava vein mosaic virus promoter (*CsVMV*) and the maize promoter (*RbcS) deposited in Addgene under the number 171715.* (**b**) Plasmid pBIOS1459 contained between the T-DNA borders (LB and RB), a neomycin resistance cassette (*EcNPTII* gene) flanked by an *Oryza sativa* (*Os*) actin promoter with its first intron and 3'Nos terminator (3'Nos). This selectable marker cassette was flanked by 2 DS elements (5'Ac and 3'Ac) and two copies of the *Gln1-3* full length cDNA with their 3' non coding regions, one flanked by the CsVMV promoter fused to the rice actin1 first intron. In addition, there was one copy of the 3'Nos terminator (3'Nos), one flanked by the promoter of the maize Rubisco small subunit (*ZmRbcS*) and one copy of the 3'Nos terminator (3'Nos).

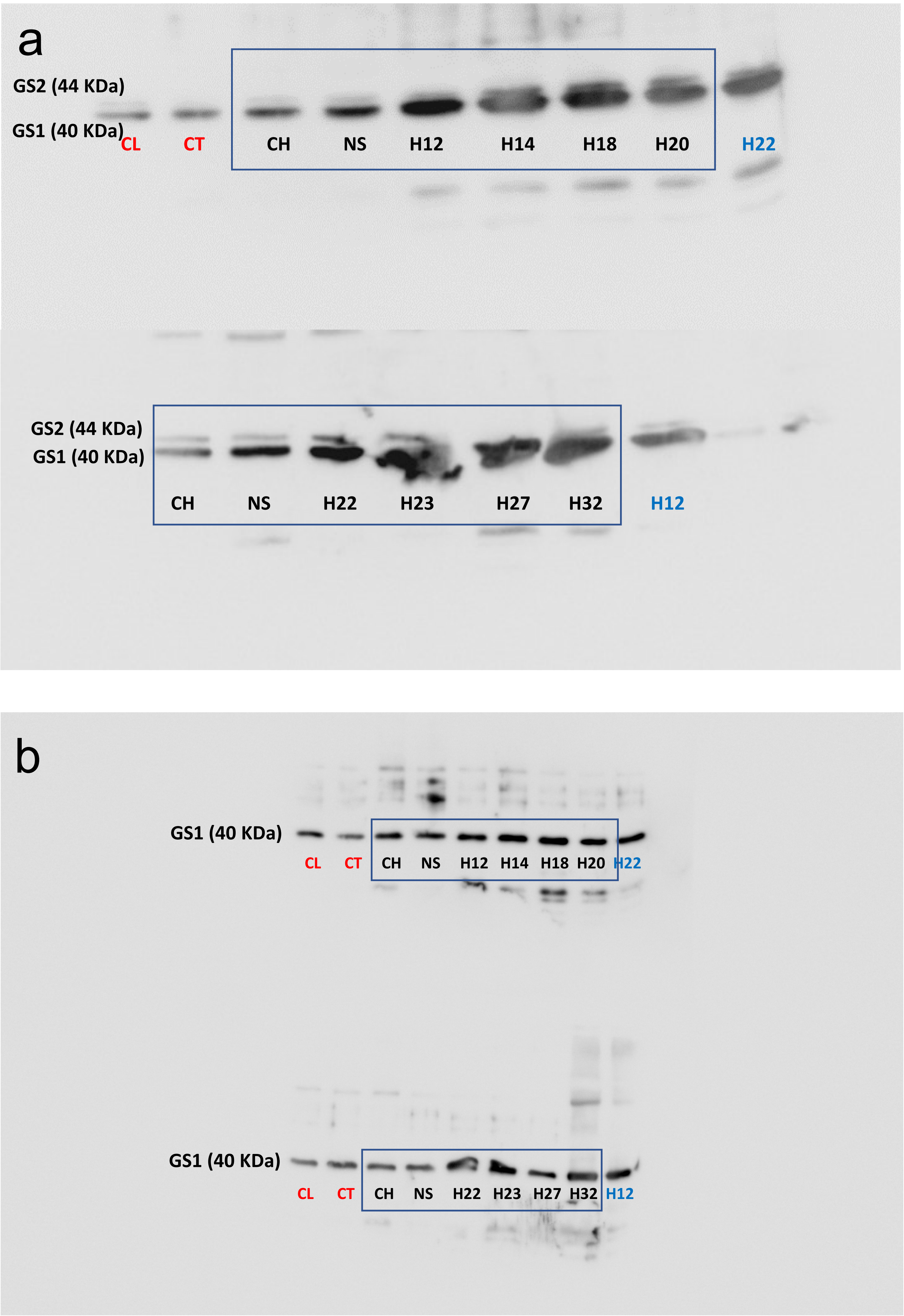

Supplementary Data Fig. 2 : Original protein gel blot analysis of the GS subunit composition in the leaves of the untransformed hybrids (CH) and null segregants (NS) and the 8 hybrids overexpressing *Gln1-3* (H12, H14, H18, H20, H22, H23, H27, H32) shown in Fig.1a and1b. Tobacco GS antibodies were used for the detection the enzymes subunits (Hirel et al., 1984). (**a**) Vegetative stage (V). (**b**) 15 days after silking (15DAS). The rectangle indicates those selected for Fig. 1. H22 and H12 in blue font are repetitions of one sample for each blot. CL and CT which are not shown on Fig. 1, represent the GS subunit composition of the untransformed control line and the tester line (RBO1) that were used to produce the hybrids. These two lines exhibits a similar GS subunit composition compared to the control hybrids (CH) and null segregants (NS). The upper band (molecular mass of 44 kDa) corresponds to the plastidic GS (GS2) subunit, and the lower band (molecular mass of 40 kDa) corresponds to the cytosolic GS (GS1) subunit. Note that GS2 subunit was not detected 15DAS (Martin et al., 2006) .

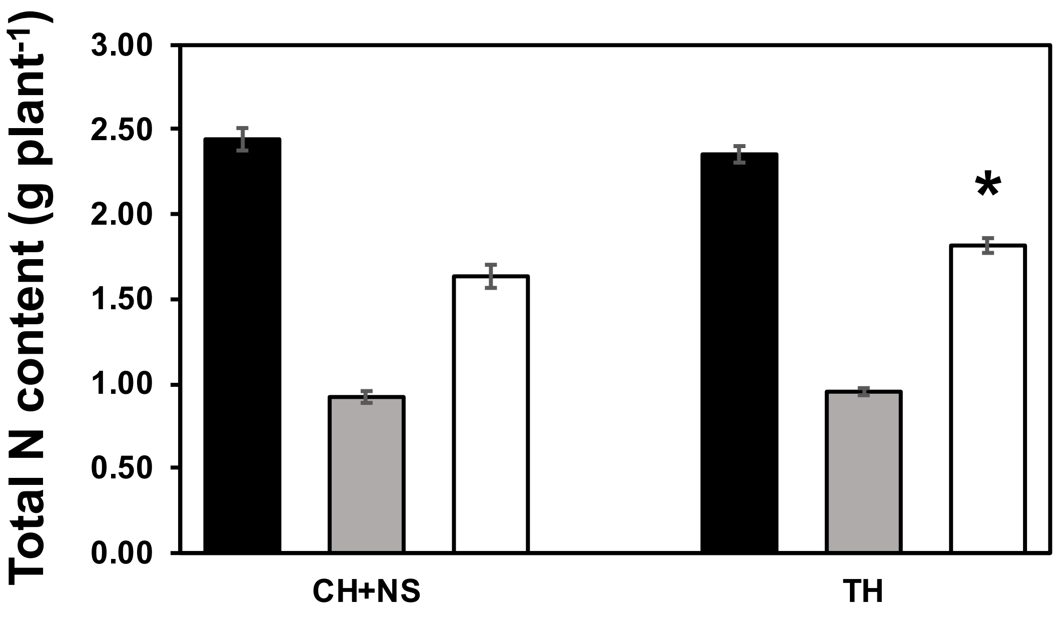

Supplementary Data Fig. 3: |Concentration of nitrogen in maize hybrids overexpressing *Gln1-3*. The hybrids were grown in the field in 2011 (Finch, M, USA). Total N was quantified in plants harvested 15DAS and at maturity. Total leaf N concentration was measured on 4 different plants for each of the 8 independent transgenic hybrids (H12 to H32). Values are the mean of the results obtained in the transgenic hybrids (TH), the untransformed hybrids (CH) and the null segregants (NS) ±SE. ■ leaf 15DAS, ■ leaf at maturity, □ kernels. The asterisk indicates a significant change ($p < 0.05$) compared to the WT and the null segregants

.

a

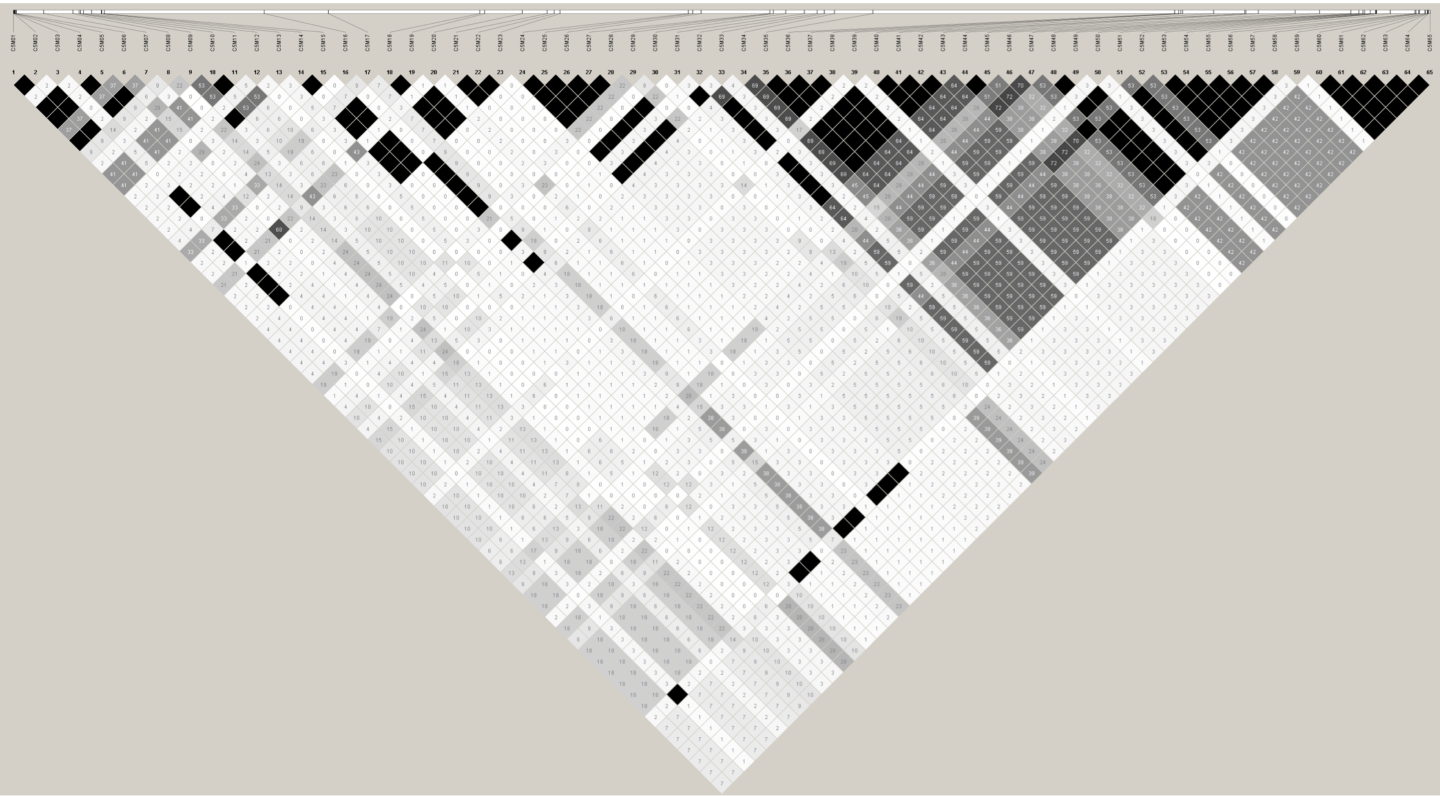

b

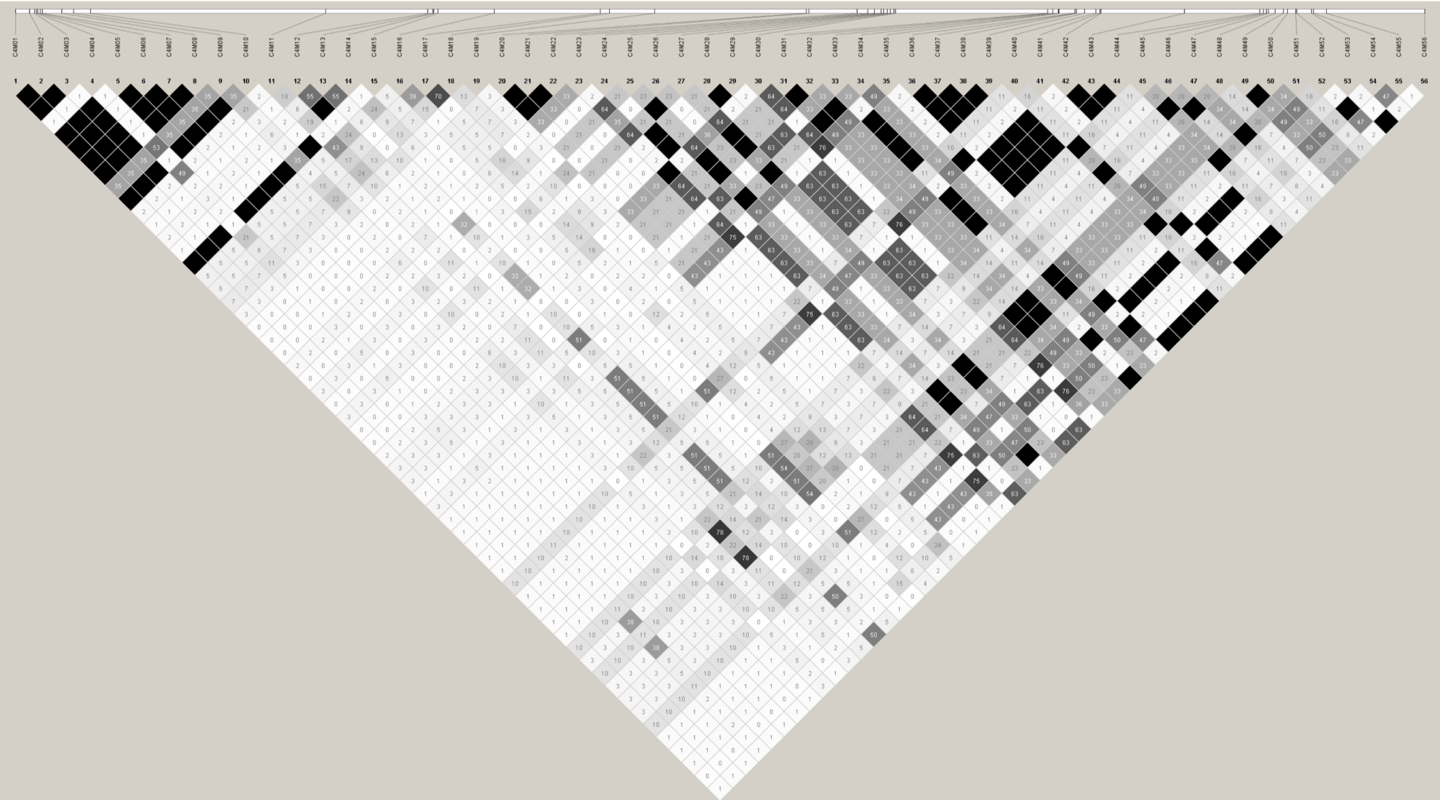

Supplementary Data Fig. 4: SNP LD plots for *Gln1-3* and *Gln1-4*. (**a**) Pairwise linkage disequilibrium (LD) plot of 65 SNPs from the maize gene encoding *Gln1.3* and flanking regions showing local strong LD at this locus on chromosome 5. (**b**) pairwise LD plot of 56 SNPs from maize *Gln1-4* gene and flanking regions shows local strong LD at this locus on chromosome 4. Each cell represents pairwise LD (r^2) between 2 SNPs, with a color code as follows: scales of grey for r2 values between 0 to 1, and black with r^2=1. Above the LD plot, the chromosomal location (Maize B73 RefgenV4, Jiao, Y. *et al.* Improved maize reference genome with single-molecule technologies. *Nature* **546**, 524–527 (2017).) of SNP markers is shown on a continuous line from left to right. The software Haploview 4.2 was used to build the matrix (58. Barrett, J. C., Fry, B., Maller, J., & Daly, M. J. Haploview: analysis and visualization of LD and haplotype maps. *Bioinformatics* **21**, 263-265 (2005).

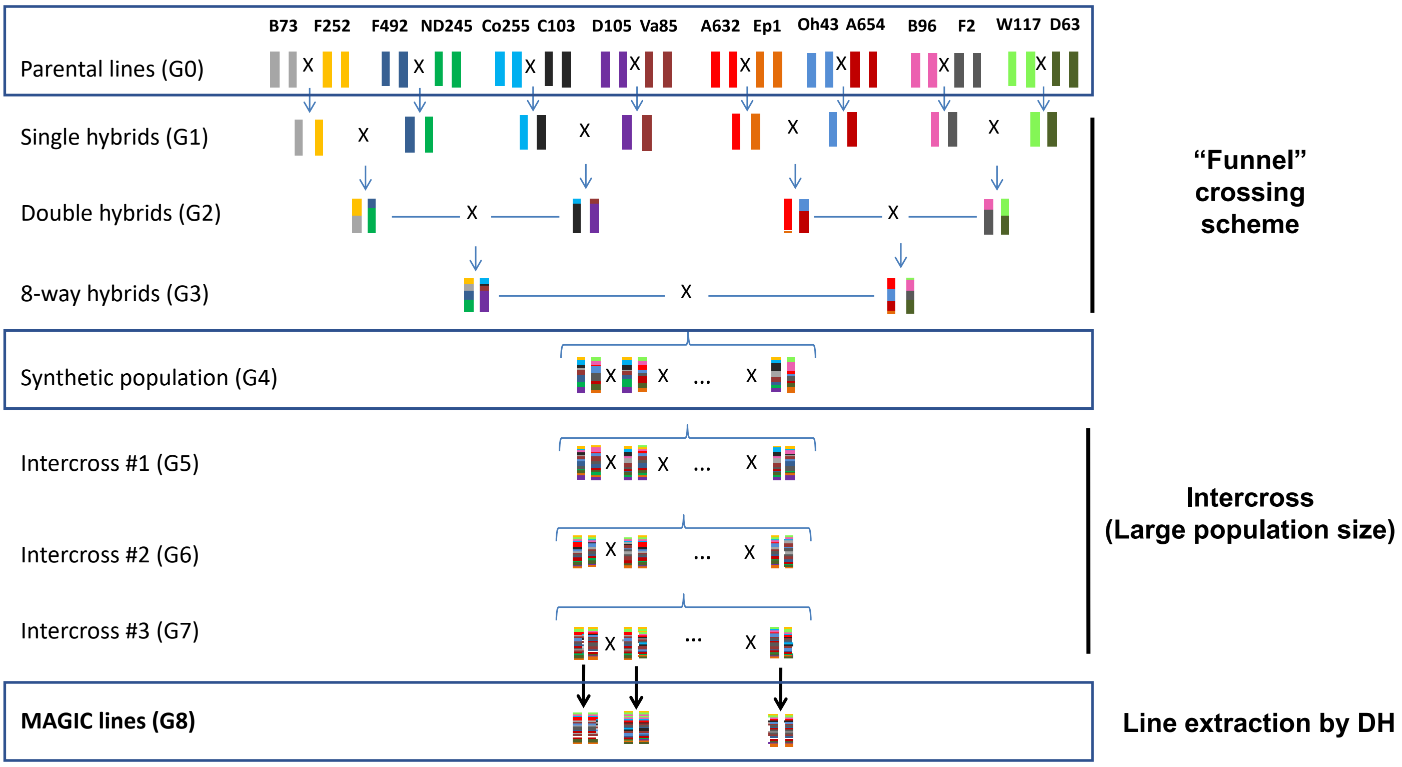

Supplementary Data Fig. 5: Production of the MAGIC panel . The MAGIC panel is issued from the crossings of 16 historical lines from the most significant heterotic groups used for hybrid production in temperate regions. At the 3rd generation of intercrossing, 543 lines were extracted by haplo-diploidization. Lines were genotyped using the Illumina Maize SNP50 bead chip and the Affymetrix Axiom Maize Genotyped Array (600K SNP). Founder lines were sequenced and 17M SNP were identified. A subset of 375 lines was extracted to remove redundant lines at the molecular level. This panel has been crossed with a common iodent tester (MBS847) so all phenotypic measurements are performed in hybrids. The range of flowering time has been restricted to one week, in order to be able to decipher complex traits as NUE, minimizing the effect on phenology on these traits. The MAGIC panel is characterized by a large genetic diversity, the absence of internal genetic structure and low linkage disequilibrium (especially near the telomeres) and there is no evidence of genetic drift meaning that each founder line on average contributes equally to the MAGIC lines.

Extended Data Table 1. Quantification of GS protein in a leaf section

	Number of gold particles. μm^{2-1}				
	Mesophyll cells		Bundle sheath cells		Companion cells
	Plastid	Cytosol	Plastid	Cytosol	Cytosol
CH	28.3 ± 3.3	14.2 ± 5.9	39.2 ± 6.9	21.2 ± 3.6	14.8 ± 4.9
H27	30 ± 14.1	50.5 ± 36*	48.2 ± 11	63.3 ± 24*	28.5 ± 6.2*
P-value	*0.8*	*0.01*	*0.067*	*0.01*	*0.014*

Immunolocalization of the enzyme was performed using transmission electron microscopy (TEM) on leaves at the V stage. Values are the mean ± SD of gold particles counted on 10-15 different sections. *Significantly different in the *Gnl1-3* overexpressor (hybrid H27) compared to the untransformed control hybrid (CH) at 0.05 probability level using the Student statistical test.
