# Supplementary material for: Impacts of environmental conditions, and allelic variation of cytosolic glutamine synthetase on maize hybrid kernel production
Source: Commun Biol. 2021 Sep 17;4:1095. doi: 10.1038/s42003-021-02598-w (PMC8448750; doi:10.1038/s42003-021-02598-w)
Supplement: Supplementary file 3 — Description of Additional Supplementary Files [file 42003_2021_2598_MOESM3_ESM.pdf]

Description of Additional Supplementary Files

File name: Supplementary Data 1

Description:

Supplementary Data 1a. List of independent transgenic hybrids used for the biochemical and physiological characterization of the Gln1-3 overexpressors and to perform field trials from 2010 to 2015.

Supplementary Data 1b. Locations of the field trials performed from 2010 to 2015 to measure yield in the hybrids overexpressing GS1 and in the controls. Yearly average temperatures are also indicated. The significant increase in kernel yield in a location is indicated in red (see Figure 4 for the corresponding data)

File name: Supplementary Data 2

Description:

Sheet*: *Raw data for biomass components. Database containing the values of the four replicates for the different agronomic traits measured in the untransformed hybrids (CH), in the two bulks of hybrid null segregants (NS) and in the hybrids overexpressing *Gln1-3* (H12 to H32). Kernel mass, DW and total N content are expressed in g/plant.

Sheet: Statistics biomass components. t-test analysis for the difference in kernel yield and biomass components between the untransformed hybrids (CH), the two bulks of hybrid null segregants (NS) and in the hybrids overexpressing *Gln1.3* (H12 to H32).

File name: Supplementary Data 3

Description: Incorporation of $^{15}NH_4^+$ in a young fully developed leaf at the vegetative (V) stage of plant development in the control hybrids (CH) and three hybrid lines overexpressing *Gln1-3* (H14, H22, H40). Significant P-values ($P<0.05$) are in bold. Labelling is expressed in mmol/gFW. Results are the mean values of four replicates for each hybrid line.

File name: Supplementary Data 4

Description:

Sheet: Yield raw data all years. Database containing informations on: the construct used for plant transformation, the independent transgenic maize hybrids in which *Gln1.3* was

overexpressed under the control of the CsVMV and Rubisco small subunit promoters, the untransformed hybrids and the hybrids null segregants used as controls, the year, location and experimental design for the field experiments, the presence of a moderate water deficit or of a water saturated soil and the impact of GS1 overexpression on kernel yield (Ql/Ha). with the statistical analysis.

Sheet: Statistics locations. Database containing the information used to evaluate the impact of *Gln1-3* overexpression each year and in each location. The background color of the locations and the stresses are the same as in the sheet Yield raw data all years and in Figure 3. Significant P-values (P<0.05) are in bold.

Sheet ANOVA yield all sites years. Results of the ANOVA statistical analysis grouping 14 field experiments performed from 2010 to 2015 in 11 different locations. Significant P-value (P<0.05) is in bold. The results for each year and each location are presented in Fig. 3a. Transgenic hybrids overexpressing *Gln1-3* analyzed: H12, H14, H17, H18, H20, H22, H23, H27, H31, H32, H39, H40 (see Extended data set 1). n = 426 for all the transgenic hybrids, and n = 92 for all the control hybrids in the 14 field trials (sheet Yield raw data all years)

Sheet: Yield Alleman 2010. Evaluation of the impact of GS1 overexpression on kernel yield in 2010 (location Alleman) compared to the controls and null segregants. H14 to H 32 = hybrids in which *Gln1-3* was overexpressed under the control of the CsVMV and Rubisco small subunit promoters. CH: untransformed control hybrids. NS: hybrids null segregants. Statistical analysis was performed for each event (event level) and for the pool of all the events (construct level). Significant P-value (P<0.05) are in bold.

Sheet: TKW Alleman 2010. Evaluation of the impact of GS1 overexpression on thousand kernel weight (TKW) in 2010 (location Alleman) compared to the controls and null segregants. H14 to H 32 = hybrids in which *Gln1-3* was overexpressed under the control of the CsVMV and Rubisco small subunit promoters. CH: untransformed control hybrids. NS: hybrids null segregants. Statistical analysis was performed for each event (event level) and for the pool of all the events (construct level).

Sheet: Yield per event location Year. Results of the statistical analysis grouping all the experiments performed from 2010 to 2015 on the 12 selected transgenic hybrids overexpressing *Gln1-3* (H14, H17, H18, H20, H22, H23, H27, H31, H32, H39, H40) in 14 field trials. Significant P-value (P<0.05) are in bold italics. WD corresponds to the three trials with a mild water deficit (George, Gibson 2013 and Sleepy Eyes 2015).

Sheet: Multilocal analysis all years. Results of the statistical analysis grouping all the experiments performed from 2010 to 2015 on the 4 selected transgenic hybrids overexpresing *Gln1-3* (H14, H20, H22, H32) present in the majority of the 14 field trials. Significant P-value (P<0.05) are in bold. WD corresponds to the three trials with a mild water deficit (George, Gibson 2013 and Sleepy Eyes 2015).

Sheet: ANOVA multilocal analysis. Results of the ANOVA statistical analysis grouping all the experiments performed from 2010 to 2015 on the 4 selected transgenic hybrids overexpressing *Gln1-3* (H14, H20, H22, H32) present in the majority of the 14 field trials. Significant P-value (P<0.05) are in bold. n = 228 for all the transgenic hybrids, and n = 92 for all the control hybrids in the 14 field trials (sheet Yield raw data all years).

Sheet: Tester interaction. Results of the statistical analysis performed in the location Sleepy eye on the 5 selected transgenic hybrids overexpressing *Gln1-3* (H14, H20, H22, H32, H39) selected for the multi-environmental filed trial analysis. The five independent transgenic events were crossed with the three testers AAX3, AAX7 and RBO1. The tester RBO1 was used to produced hybrids for all the other field trials.

File name: Supplementary Data 5

Description: Database containing parameters of the SNP association study for *Gln1-3* and *Gln1-4* genes. An example of the resulting Manhattan plots is presented in Figure 4 for Saint-Paul in 2016 and Blois in 2015 and the results of the association study in the different locations in 2015, 2016 and 2017 summarized in Table 1. "Allele1" and "Allele2" represents the SNP polymorphic sites. "Number of lines. Allele1" or "Number of lines. Allele2" represents the number of lines carrying each allele. The corresponding allele frequency is also indicated. "Mean Effect" represents the effect of all alleles taken together (for a given SNP). "Effect.Allele1" or "Effect.Allele2" are the additive effect of each allele, each compared to the mean value centered on "0". Effects are shown only for alleles with mlog10_p-values ≥2.

File name: Supplementary Data 6

Description:

Sheet Gln1-3 SNP genotyping. Database containing the MAGIC population SNP genotyping data corresponding to the gene region of *Gln1-3*. Each line corresponds to each MAGIC parents or MAGIC DHILs. Each column corresponds to each SNP marker with chromosome and position on B73 RefGen_v4 maize genome.

Sheet Gln1-3 LD values. Database containing the SNP pairwise measures of LD (r^2) in the gene region of *Gln1*-3.

Sheet Gln1-4 SNP genotyping. Database containing the MAGIC population SNP genotyping data corresponding to the gene region of Gln1-4. Each line corresponds to each MAGIC parents or MAGIC DHILs. Each column corresponds to each SNP marker with chromosome and position on the B73 RefGen_v4 maize genome.

Sheet Gln1-4 LD values. Database containing the SNP pairwise measures of LD (r^2) in the gene region of Gln1-4.

Sheet Gln1-3 Gln1-4 structure bounds. Database showing the gene structures bounds in the gene regions of *Gln1-3* and *Gln1-4*. A given Filtered Gene Set (FGS), (gene coordinate RefGen_v4, Jiao et al 2017) was extended with 5000bp (P5000) into the putative promoter region (Li et al 2012) and extended with 500bp (T500) into the 3′ terminal region, for the final selection of SNPs. Gene transcripts prediction are from AGP_v4.38 (mgdb.org/gramene.org).

File name: Supplementary Data 7

Description: Adjusted mean values for kernel yield at 15% moisture obtained with the hybrids of the MAGIC population grown in the field in 2015, 2016 and 2017 in three different locations (Blois, Saint-Paul and Nerac). Results are expressed in Ql/ha. NA = samples not available.

File name: Supplementary Data 8

Description: Supplementary Data for Fig1.

File name: Supplementary Data 9

Description: Supplementary Data for Fig. 3.

File name: Supplementary Data 10

Description: Supplementary Data for Fig. 4.
