# Supplementary material for: Impacts of environmental conditions, and allelic variation of cytosolic glutamine synthetase on maize hybrid kernel production
Source: Commun Biol. 2021 Sep 17;4:1095. doi: 10.1038/s42003-021-02598-w (PMC8448750; doi:10.1038/s42003-021-02598-w)
Supplement: Supplementary file 14 — Reporting Summary [file 42003_2021_2598_MOESM14_ESM.pdf]

nature research

Corresponding author(s): Bertrand HIREL
COMMSBIO-20-2908-A

Last updated by author(s): May 27, 2021

Reporting Summary

Nature Research wishes to improve the reproducibility of the work that we publish. This form provides structure for consistency and transparency in reporting. For further information on Nature Research policies, see our Editorial Policies and the Editorial Policy Checklist.

Statistics

For all statistical analyses, confirm that the following items are present in the figure legend, table legend, main text, or Methods section.

n/a	Confirmed	
☐	☒	The exact sample size (n) for each experimental group/condition, given as a discrete number and unit of measurement
☐	☒	A statement on whether measurements were taken from distinct samples or whether the same sample was measured repeatedly
☐	☒	The statistical test(s) used AND whether they are one- or two-sided<br>*Only common tests should be described solely by name; describe more complex techniques in the Methods section.*
☐	☒	A description of all covariates tested
☒	☐	A description of any assumptions or corrections, such as tests of normality and adjustment for multiple comparisons
☐	☒	A full description of the statistical parameters including central tendency (e.g. means) or other basic estimates (e.g. regression coefficient) AND variation (e.g. standard deviation) or associated estimates of uncertainty (e.g. confidence intervals)
☐	☒	For null hypothesis testing, the test statistic (e.g. F, t, r) with confidence intervals, effect sizes, degrees of freedom and P value noted<br>*Give P values as exact values whenever suitable.*
☒	☐	For Bayesian analysis, information on the choice of priors and Markov chain Monte Carlo settings
☐	☒	For hierarchical and complex designs, identification of the appropriate level for tests and full reporting of outcomes
☒	☐	Estimates of effect sizes (e.g. Cohen's d, Pearson's r), indicating how they were calculated

Our web collection on statistics for biologists contains articles on many of the points above.

Software and code

Policy information about availability of computer code

Data collection	Not applicable
Data analysis	Not applicable

For manuscripts utilizing custom algorithms or software that are central to the research but not yet described in published literature, software must be made available to editors and reviewers. We strongly encourage code deposition in a community repository (e.g. GitHub). See the Nature Research guidelines for submitting code & software for further information.

Data

Policy information about availability of data

All manuscripts must include a data availability statement. This statement should provide the following information, where applicable:

- Accession codes, unique identifiers, or web links for publicly available datasets
- A list of figures that have associated raw data
- A description of any restrictions on data availability

A list of supplemntary raw data associated to the figures is provided (Extended data sets 1 to 7). These data will be availble withouht any restrictions.

Field-specific reporting

Life sciences study design

All studies must disclose on these points even when the disclosure is negative.

Sample size	For the selection and characterization of the transgenic hybrids overexpressing the enzyme glutamine synthetase eight independent transgenic events were used which strongly limits the possibility that the insertion of the transgene had any major impact on plant phenotypic traits including physiological, biochemical, and agronomic ones.<br>In addition, the number of independent transgenic events for the maize hybrids tested in the field for kernel production was comprised between 8 to 10 (see extended data set 1) which is also far above that usually used to study the impact of a genetic manipulation on plant performances both in model and crop species (around 3 to four in most papers published in the field). Although part of these hybrids was not tested over the five years of experimentation depending on seed availability, four of them were repeatedly used to obtained data when plants were grown in different locations and over the five years of experimentation.<br>For each field trial, sample size was determined to ensure that there was a sufficient number of repetitions to observe significant differences in kernel production. To achieve this, the control and transgenic hybrids were grown in the field in a random block design, completely balanced lattice or split plot depending on the year of experimentation and on the location, with four to five replicates for each control and transgenic hybrid line (see extended data set 4). An outside border area of at least six rows was planted surrounding the different plots of the field trial to avoid as much as possible side effects. Each plot was composed of two rows of maize of 6m in length. The number of plants in each plot was between 60 and 75 depending on the location (corresponding to classical planting density in the cornbelt), which is optimal to measure maize kernel yield and its components in a reproducible manner and to further perform statistical analysis in a reliable manner<br>The MAGIC association panel size was predetermined to be sufficient for association study with this type of genetic material. Power of the experiment has been assessed based on simulation, at 95% probability to detect a QTL explaining 5% of phenotypic variation (pvalue=0.001).
Data exclusions	There was no data exclusion both in the field trials with the transgenic hybrids and in the the association genetics study.
Replication	During the field experiment performed in 2011 samples were harvested to characterise the 8 independent transgenic hybrids both at the biochemical and agronomic levels. This allowed to perform one-way ANOVA statistical analyses using a Student-Newman-Keuls test (t-test) to determine when the differences were significant.<br>For all the other field experiments performed from 2010 to 2015 to measure differences in yield between the controls and the transgenic hybrids, a two-way statistical analysis was performed used a mixed model restricted maximum likelihood (REML). The model is the following: Random factors = replicate (complete), sub-block (ibloc) tested in the replicate (for lattice only), Fixed factor = construct. A test of the differences in terms of the least square means was made for the fixed effects at the construct level between the control hybrids (CH and NS) and the transgenic hybrids.<br>Such experimental design allowed to determine when the differences in kernel production between control and transgenic hybrids were significant. When these differences where not significant, it was clearly stated in the results section and then critically discussed notably as a function of the environmental conditions.<br>The field experiments for the association genetics study were successfully reproduced in three different locations and over three years for Gln1-3 and two different locations in 2015 for Gln1-4 (p-Value>2). Heritabilities of the gentic means were 0.65 for kernel yield at location Blois in 2015, 0.84 at Saint-Paul in 2015, 0.81 at Saint-Paul in 2016, 0.86 at Blois in 2017 and 0.84 at Nerac in 2017.
Randomization	In the field experiments performed over five years and in the different locations in the USA, the control and transgenic hybrids were grown in a random block design, completely balanced lattice or split plot depending on the year of experimentation and on the location, with four to five replicates for each hybrid line (see extended data set 4).<br>The field experiments for the MAGIC association panel were carried out following an alpha lattice design with two replicates for each trial.
Blinding	There was no blinded group allocation.

Reporting for specific materials, systems and methods

We require information from authors about some types of materials, experimental systems and methods used in many studies. Here, indicate whether each material, system or method listed is relevant to your study. If you are not sure if a list item applies to your research, read the appropriate section before selecting a response.

Materials & experimental systems

n/a	Involved in the study	
☐	☒	Antibodies
☒	☐	Eukaryotic cell lines
☒	☐	Palaeontology and archaeology
☒	☐	Animals and other organisms
☒	☐	Human research participants
☒	☐	Clinical data
☒	☐	Dual use research of concern

Methods

n/a	Involved in the study	
☒	☐	ChIP-seq
☒	☐	Flow cytometry
☒	☐	MRI-based neuroimaging

Antibodies

Antibodies used	Antibodies against the plastidic form of glutamine synthetase (Hirel et al.,1982 - Glutamine synthetase in spinach leaves. Immunological studies and immuno-cytochemical localization. Plant Physiol. 69 : 983-987).
Validation	Validation in maize for immunodection of plastidic (GS2) and cytosolic gs (GS1) published by: Becker et al., 1993. - Subcellular and immunocytochemical localization of the enzymes involved in ammonia assimilation in mesophyll and bundle sheath strands of maize leaves. Planta 191 : 129-136.
